# Astragalus Extract Mixture HT042 Improves Bone Growth, Mass, and Microarchitecture in Prepubertal Female Rats: A Microcomputed Tomographic Study

**DOI:** 10.1155/2017/5219418

**Published:** 2017-05-10

**Authors:** Jungbin Song, Sung Hyun Lee, Donghun Lee, Hocheol Kim

**Affiliations:** ^1^Department of Herbal Pharmacology, College of Korean Medicine, Kyung Hee University, 26 Kyungheedae-ro, Dongdaemun-gu, Seoul 02447, Republic of Korea; ^2^Korea Institute of Science and Technology for Eastern Medicine (KISTEM), NeuMed Inc., 88 Imun-ro, Dongdaemun-gu, Seoul 02440, Republic of Korea

## Abstract

Astragalus extract mixture HT042 is a standardized multiherbal mixture comprising* Astragalus membranaceus*,* Eleutherococcus senticosus,* and* Phlomis umbrosa*, which has proven to promote children's height growth. The aim of this study was to investigate the effects of HT042 on longitudinal bone growth, bone mass, and bone microstructure in growing rats using a high-resolution microcomputed tomography system. Four-week-old female rats were fed an HT042-containing diet for 2 weeks. Tibial length was measured at baseline and weekly* in vivo*. At the end of the study, volumetric bone mineral density (vBMD) and microarchitectural parameters were estimated in the trabecular and cortical bone of the tibia. Tibial length gain was significantly increased by HT042 compared to that reported with the control diet. In the proximal tibial metaphysis, HT042-treated rats had significantly higher trabecular vBMD, bone volume fraction, and trabecular number and lower trabecular separation, trabecular pattern factor, and structure model index values than control rats did. Total cross-sectional area and bone area of the cortical bone in the tibial diaphysis also increased. These findings suggest that HT042 increases longitudinal bone growth rate, improves trabecular bone mass, and enhances the microarchitecture of trabecular and cortical bone during growth.

## 1. Introduction

One of the main functions of bone is to provide structural support for the body. During childhood, bones grow in size, accrue mass, and change their architecture to develop a strong structure for load bearing [1]. The percentile location of an individual's bone traits, such as bone mineral density and trabecular and cortical morphology, all of which determine bone strength, is likely established during childhood [2–5]. Therefore, the optimization of bone strength during childhood is important for lifelong bone health.

Several studies have demonstrated that short-statured children have impaired bone health. Low bone mineral content (BMC) and bone mineral density (BMD) at several skeletal sites are reported in short children [6–9]. Short children exhibit increased bone resorption [6] and have impaired bone structure and bone strength [10]. Growth hormone (GH) treatment has been reported to increase height, normalize BMD, and improve bone structure and strength in short children [6, 9, 10]. In addition to well-established effects on growth plate cartilage, GH exerts anabolic effects on bone and stimulates new bone formation, which results in greater bone mass and improved skeletal structure [11, 12].

Astragalus extract mixture HT042 is a standardized multiherbal mixture consisting of* Astragalus membranaceus*,* Eleutherococcus senticosus*, and* Phlomis umbrosa*. HT042 was found to increase height growth rate in children with mild short stature in a 12-week placebo-controlled trial, which was confirmed by another 24-week trial (data not published). HT042 has been shown to induce longitudinal bone growth by stimulation of chondrocyte proliferation and hypertrophy in growth plates [13, 14]. The mechanism underlying HT042-induced bone growth involves the stimulation of GH secretion [15], which is assumed to lead to the systemic and local production of insulin-like growth factor-1 (IGF-1) [13, 14]. This mechanism was also supported by a previous report where* A. membranaceus* induced GH release [16]. HT042 has been suggested to stimulate GH secretion; therefore, we hypothesized that HT042 increases longitudinal bone growth and simultaneously enhances bone mass and microarchitecture during growth.

To address this hypothesis, we adopted a high-resolution microcomputed tomography (*μ*-CT) system. Four-week-old female Sprague-Dawley rats were fed a diet that contained HT042 for 2 weeks. This treatment period was chosen because it corresponds with the human childhood period between the approximate ages of five and nine years [17]. The length of the tibia was measured at baseline and then weekly for 2 weeks using* in vivo μ*-CT. In comparison with our previous study using densitometry [14], we achieved more accurate three-dimensional (3D) analysis of bone length in a nondestructive way. Volumetric BMD and microarchitectural changes in the trabecular and cortical bone of the tibia were measured after 2 weeks using* ex vivo μ*-CT.

## 2. Materials and Methods

### 2.1. Sample Preparation and HPLC Analysis

HT042 consists of the extracts of three medicinal plants: the roots of* A. membranaceus*, the stems of* E. senticosus*, and the roots of* P. umbrosa*. It is standardized to contain 0.008% formononetin, 0.36% eleutheroside E, and 0.15% shanzhiside methyl ester. HT042, manufactured in accordance with the process registered by the Korean Ministry of Food and Drug Safety (MFDS), was purchased from NeuMed Inc. (Seoul, Korea). For quality assessment, the contents of marker compounds were quantified with high performance liquid chromatography (HPLC). HPLC analysis was performed on a Waters instrument (Milford, MA, USA) equipped with a Waters 1525 pump, a Waters 2707 autosampler, and a Waters 2998 photodiode array detector. A reverse-phase SunFire™ C18 column (250 × 4.6 mm i.d., 5 *μ*m particle size, Waters) was used and kept at 40°C. The mobile phase consisting of 0.5% phosphoric acid (A) and acetonitrile (B) was used at a flow rate of 1.0 mL/min. The gradient elution conditions for detection were as follows: formononetin, 0-15-25-28-30 min, 35-35-65-35-35% solvent B; eleutheroside E and shanzhiside methyl ester, 0-20-30-40-45 min, 5-17-22-30-5% solvent B. Formononetin, eleutheroside E, and shanzhiside methyl ester were monitored at 245 nm, 210 nm, and 235 nm, respectively.

### 2.2. Animals and Diets

Twenty-one-day-old female Sprague-Dawley rats (weight: 45 ± 5 g) were obtained from Samtako (Osan, Korea). The rats were housed under controlled conditions of temperature (23 ± 1°C), relative humidity (55 ± 5%), and lighting (07:00–19:00 h). After 7 days of acclimatization, the rats were randomly divided into four groups with nine rats in each group: control, GH (positive control), 0.2% HT042, and 0.6% HT042. The control group received control chow only for 2 weeks. The 0.2% and 0.6% HT042 groups received chow containing 0.2% and 0.6% HT042, which were equivalent to approximately 200 and 600 mg/kg/day HT042, respectively. The GH group received control chow and subcutaneous injections of 200 *μ*g/kg recombinant human growth hormone (Eutropin®, LG Life Sciences, Seoul, Korea) once daily for 2 weeks. All rats were provided with* ad libitum* access to distilled water throughout the experiment. Body weight and food intake were measured daily. At the end of the 2-week study, all rats were sacrificed and the tibiae were collected from each rat for further *μ*-CT analysis. All experimental procedures were performed in accordance with the guidelines of the Institutional Animal Care and Use Committee of Korea Institute of Science and Technology for Eastern Medicine (KISTEM) (protocol number KISTEM-IACUC-2016-001).

### 2.3. Microcomputed Tomography Analysis

The bone length, mineral density, and microarchitecture of tibia were assessed using *μ*-CT (SkyScan1176, Skyscan, Belgium). The X-ray source was set at an energy of 50 kV and intensity of 200 *μ*A, with a pixel size of 8.9 *μ*m. Samples were scanned through a 180° rotation angle with rotation steps of 0.8° (*in vivo*) and 0.4° (*ex vivo*).

To monitor the changes in tibial length, whole tibiae were scanned* in vivo* at the beginning of the study and at days 7 and 14 under isoflurane anesthesia. The images were reconstructed using NRecon software (Skyscan v. 1.6.10.1). The tibial length was measured as the distance between the proximal aspect of the head of the tibia and the most distal aspect of the medial malleolus using DataViewer software (Skyscan v.1.5.1.9).

Volumetric bone mineral density (vBMD) and bone microstructure were analyzed in the trabecular and cortical bone of the tibia.* Ex vivo* scanning was performed using a 0.5 mm thick aluminum filter. The volume of interest (VOI) for the trabecular and cortical bone was defined as the region beginning 0.43 mm and 4.78 mm distally from the proximal growth plate, respectively. A total of 450 slices were analyzed for the trabecular bone and 100 slices for the cortical bone (8.69 *μ*m/slice). For vBMD measurements, the calibration was performed by scanning a phantom with known vBMD (0.25 and 0.75 g/cm^3^). The trabecular microarchitecture parameters include trabecular bone volume per unit of total volume (BV/TV, bone volume fraction), trabecular thickness (Tb.Th), trabecular number (Tb.N), trabecular separation (Tb.Sp), trabecular pattern factor (Tb.Pf), and structure model index (SMI). The cortical indices included total cross-sectional area (Tt.Ar), cortical bone area (Ct.Ar), medullary area (Ma.Ar), cortical area fraction (Ct.Ar/Tt.Ar), and cortical thickness (Ct.Th). Raw image data were reconstructed using NRecon software (version 1.6.10.1, Skyscan) and analyzed using CT Analyser software (version 1.15.4.0, Skyscan).

### 2.4. Statistical Analysis

All statistical analyses were performed using GraphPad Prism 5 (GraphPad Software, CA, USA). Differences between groups were analyzed by one-way analysis of variance followed by Dunnett's test. Values of *p* < 0.05 were considered statistically significant. All values were expressed as the mean ± standard deviation.

## 3. Results

### 3.1. HPLC Analysis of HT042

**Figure 1:**
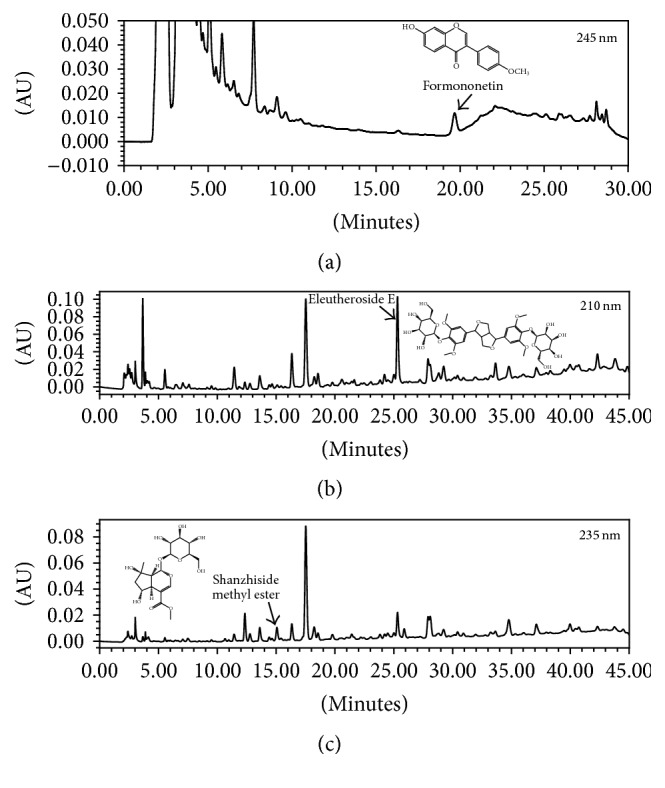
HPLC chromatograms of HT042. Arrows in (a), (b), and (c) show the peaks of formononetin, eleutheroside E, and shanzhiside methyl ester, respectively. The monitoring UV wavelength for each marker compound is shown in the top right corner of each chromatogram.

To confirm the quality of the sample used for this study, the contents of marker compounds were measured using HPLC.  Figure 1 shows the HPLC chromatograms of the three marker compounds detected in the sample. The contents of formononetin, eleutheroside E, and shanzhiside methyl ester were quantified as 91.0 *μ*g/g, 3.9 mg/g, and 1.5 mg/g, respectively.

### 3.2. Body Weight and Food Intake

As shown in Table 1, there was no difference in body weight and food intake between groups. The body weight gain during a period of 2 weeks significantly increased in the GH group compared with that in the control group (*p* < 0.05).

### 3.3. Changes in Tibial Length

**Figure 2:**
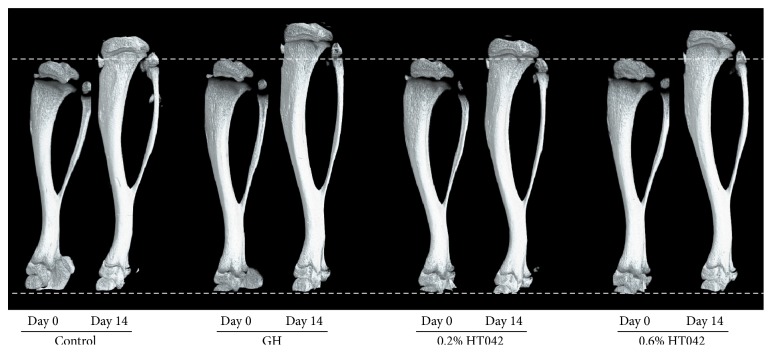
Representative images depicting 3D reconstructed tibia obtained at days 0 and 14 using *μ*-CT. The distance between the two dotted lines indicates the initial tibial length of 4-week-old rats.

The tibial length was measured at baseline and at days 7 and 14 using a *μ*-CT system. The tibial length gain during the first week significantly increased in the 0.6% HT042 group compared with that in the control group (2.92 ± 0.24 versus 2.61 ± 0.17 mm, *p* < 0.05, Table 2). This increase was comparable with that observed in the GH group. During the second week, the tibial length gain was higher in rats in the GH and 0.6% HT042 groups, but this difference was not statistically significant compared with that observed for the control rats. The cumulative increase during the period of 2 weeks was significantly greater in the 0.6% HT042 group than in the control group (4.70 ± 0.34 versus 4.20 ± 0.37 mm, *p* < 0.05).  Figure 2 shows representative 3D *μ*-CT images of the tibia obtained at days 0 and 14.

### 3.4. Volumetric Bone Mineral Density

**Figure 3:**
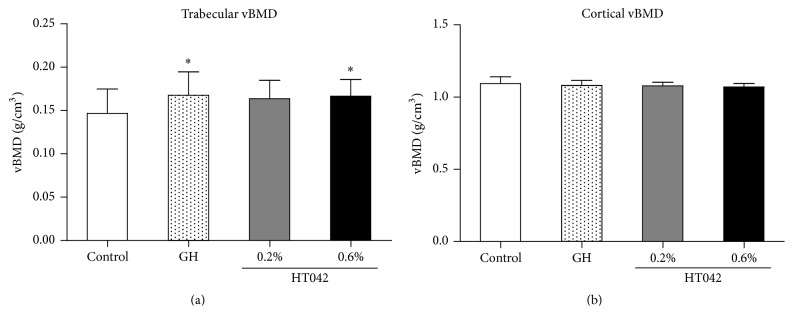
Volumetric BMD of the proximal tibia measured by *μ*-CT. (a) Trabecular vBMD of the proximal tibial metaphysis. (b) Cortical vBMD of the proximal tibial diaphysis. Values are expressed as the mean ± SD. ^*∗*^*p* < 0.05 versus control group. *n* = 7-8 per group.

In the proximal tibial metaphysis, the trabecular vBMD was 0.147 ± 0.028 g/cm^3^ in the control group (Figure 3(a)). The trabecular vBMDs of the GH group (0.168 ± 0.027 g/cm^3^) and 0.6% HT042 group (0.167 ± 0.019 g/cm^3^) were significantly higher than those of the control group (both *p* < 0.05). In contrast, there were no significant differences in the cortical vBMD between the groups (Figure 3(b)).

### 3.5. Bone Microarchitecture

**Figure 4:**
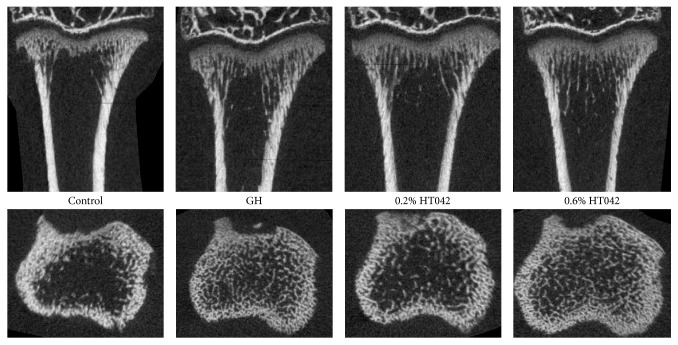
*μ*-CT images of proximal tibial metaphysis in each group. The top row represents the images of the midsagittal planes of the proximal tibial metaphysis. The bottom row represents the images of coronal planes 1.5 mm below the tibial proximal growth plate.

In the proximal tibial metaphysis, GH and HT042 resulted in significant differences in trabecular *μ*-CT parameters (Table 3). In particular, rats treated with GH or fed HT042 had greater BV/TV than the control rats did (both *p* < 0.05). The greater BV/TV was accompanied by a higher Tb.N (*p* < 0.05) and lower Tb.Sp (*p* < 0.05). The rats treated with GH or fed HT042 also had significantly lower Tb.Pf than the control rats did. HT042-fed rats had a lower SMI than the control rats did, while GH-treated rats did not. The proximal tibial metaphysis of each group under the scan of *μ*-CT is shown in Figure 4, which demonstrates the positive effect of GH and HT042 on bone microstructure formation.

Investigation of cortical bone microarchitecture revealed that rats in the GH and 0.6% HT042 groups had significantly higher Tt.Ar and Ct.Ar than the control rats did (*p* < 0.05, Table 4). There were no significant differences in Ma.Ar, Ct.Ar/Tt.Ar, and Ct.Th.

## 4. Discussion

The present findings demonstrated that the 2-week administration of the HT042 diet increased the growth rate of tibial length in prepubertal female rats. In the proximal tibial metaphysis, HT042 resulted in increased trabecular vBMD, BV/TV, and Tb.N and decreased Tb.Sp, Tb.Pf, and SMI values. HT042 also increased the total cross-sectional area and bone area of the cortical bone in the tibial diaphysis.

The tibial length gain over 2 weeks was significantly greater, by 11.8%, in rats fed the 0.6% HT042 diet compared with that in the control rats. The tibial growth rate in the control group was 372.2 and 228.0 *μ*m/day during the first and second weeks, respectively, which corresponded with the previous observation [18, 19]. HT042 was shown to increase the tibial growth rate, which confirmed our previous studies on linear bone growth [13, 14]. It was noteworthy that the growth rate reached levels that were comparable to those induced by GH. The results suggest that HT042 increases longitudinal bone growth rate.

Rats fed a diet containing 0.6% HT042 had significantly higher trabecular vBMD and bone volume fraction, of 13.6% and 26.5%, respectively, than control rats did, which indicated a higher trabecular bone mass. During growth, the accumulation of trabecular bone mass in the metaphyseal region mostly results from longitudinal bone growth [19]. Chondrocytes within growth plate cartilage contribute to longitudinal bone growth through a combination of proliferation, cartilage matrix synthesis, and hypertrophy [20]. The hypertrophic chondrocytes mineralize the cartilage matrix by secreting matrix vesicles and then osteoblasts deposit bone matrix on the remaining cartilage, resulting in trabecular bone formation [21]. During the process of longitudinal bone growth, HT042 stimulates the proliferation and hypertrophy of chondrocytes, which result in increased growth plate height [13, 14]. The mechanism underlying HT042-induced linear growth involves the stimulation of GH secretion and subsequent production of systemic and local IGF-1 [13–15]. Although GH is mostly known for its effects on growth plate cartilage, it also stimulates osteoblast proliferation and activity, thereby increasing trabecular bone formation [22, 23]. The HT042-induced increase in trabecular bone mass may result from improved longitudinal bone growth and bone formation, which was possibly caused by the stimulation of GH release.

Adequate bone mass and microarchitecture allow bones to be stronger and highly resistant to fracture [24]. HT042 led to notable alterations in the trabecular bone microarchitecture reflected by higher Tb.N and lower Tb.Sp, Tb.Pf, and SMI values that were comparable with the results obtained with GH treatment. Higher Tb.N and lower Tb.Sp indicate that HT042 increases trabecular bone mass as a result of trabecular formation, as well as by causing a decrease in trabecular separation. Both HT042 and GH had no effects on Tb.Th. In line with our data, it was previously reported that GH treatment did not show an increase in Tb.Th in growth hormone-deficient mice [25]. Three-dimensional simulations of bone loss have revealed that an increase in the trabecular number is more important for trabecular bone strength than an increase in the trabecular thickness [26], which could possibly translate to the improved mechanical competence of the trabecular bone in HT042-fed rats compared with control rats. A lower Tb.Pf value indicates better connected trabecular lattices [27]. As an increase in trabecular connectivity is associated with increased bone strength [28], the decreased Tb.Pf in the HT042 groups indicates that HT042 could exert positive effects on the mechanical strength of bone. SMI is an indicator of the relative prevalence of plates and rods in the trabecular bone [29]. Lower SMI values reflect a shift from rod-like to plate-like structure, a transition that typically enhances bone strength [30, 31]. As SMI is a strong predictor of bone mechanical behavior [32], HT042-induced changes could have important consequences for the improvement of bone strength. Collectively, these results suggest that HT042 improves trabecular bone microarchitecture and may therefore enhance bone strength.

HT042 also resulted in alterations to the cortical bone microarchitecture. HT042 led to increased Tt.Ar and Ct.Ar but did not change Ma.Ar, which suggests that HT042 induces periosteal bone apposition. As the cortical area is one of the key predictors of bone strength and fracture resistance [33], these results support the beneficial effects of HT042 on bone health.

In our study, cortical vBMD and thickness were not altered by either HT042 or GH treatment. In contrast to our findings, Sundström et al. [34] demonstrated that GH injection increased BMC and the thickness of cortical bone in normal growing rats. In this study, GH was subcutaneously injected twice daily at 5 mg/kg/day for 28 days, whereas, in our study, it was injected once daily at 200 *μ*g/kg/day for 14 days. It is possible that the dose and duration of the treatment chosen for this study were not sufficient to be biologically active. As HT042 has been suggested to stimulate GH secretion [15], it is premature to conclude that HT042 does not increase cortical bone mass or thickness. However, further studies are needed to address this issue.

## 5. Conclusion

In summary, HT042 increases longitudinal bone growth rate, improves trabecular bone mass, and enhances the microarchitecture of trabecular and cortical bone during growth. Based on these findings, it may be concluded that HT042 is beneficial, not only for the promotion of the growth of short-statured children, but also, more importantly, for the enhancement of bone strength and quality during the growth stage.

## Figures and Tables

**Table 1:** Body weight and food intake.

	Control	GH	0.2% HT042	0.6% HT042
Initial body weight (g)	88.0 ± 6.6	86.1 ± 5.4	88.7 ± 4.3	87.0 ± 6.0
Final body weight (g)	140.5 ± 9.4	149.1 ± 12.5	148.6 ± 12.5	148.9 ± 7.2
Body weight gain (g)	52.5 ± 6.5	63.0 ± 7.9^*∗*^	59.9 ± 9.2	61.9 ± 5.1
Food intake (g/rat/day)	12.5 ± 0.6	12.8 ± 0.3	13.3 ± 0.7	13.0 ± 0.8

Values are expressed as the mean ± SD. ^*∗*^*p* < 0.05  versus control group. *n* = 7-8 per group.

**Table 2:** The gain in tibial length (mm) during 2-week administration of HT042 diet.

	Control	GH	0.2% HT042	0.6% HT042
Days 0–7	2.61 ± 0.17	2.87 ± 0.13^*∗*^	2.78 ± 0.18	2.92 ± 0.24^*∗∗*^
Days 7–14	1.60 ± 0.32	1.89 ± 0.18	1.69 ± 0.33	1.78 ± 0.26
Days 0–14	4.20 ± 0.37	4.75 ± 0.23^*∗∗*^	4.48 ± 0.24	4.70 ± 0.34^*∗*^

Values are expressed as the mean ± SD. ^*∗*^*p* < 0.05 and ^*∗∗*^*p* < 0.01  versus control group. *n* = 7-8 per group.

**Table 3:** Trabecular bone microarchitecture in the proximal tibial metaphysis.

Parameters (unit)	Control	GH	0.2% HT042	0.6% HT042
BV/TV (%)	19.018 ± 4.961	24.001 ± 5.257^*∗*^	22.973 ± 4.145	24.053 ± 4.589^*∗*^
Tb.Th (mm)	0.092 ± 0.005	0.094 ± 0.005	0.091 ± 0.005	0.093 ± 0.004
Tb.N (mm^−1^)	2.073 ± 0.575	2.570 ±0.575^*∗*^	2.535 ± 0.523	2.581 ± 0.501^*∗*^
Tb.Sp (mm)	0.705 ± 0.211	0.543 ± 0.119^*∗*^	0.544 ± 0.128^*∗*^	0.560 ± 0.133^*∗*^
Tb.Pf (mm^−1^)	10.847 ± 3.491	7.936 ± 2.873^*∗*^	8.224 ± 2.523^*∗*^	7.413 ± 2.248^*∗∗*^
SMI	1.833 ± 0.264	1.638 ± 0.237	1.623 ± 0.212^*∗*^	1.602 ± 0.195^*∗*^

Values are expressed as the mean ± SD. ^*∗*^*p* < 0.05 and ^*∗∗*^*p* < 0.01  versus control group. *n* = 7-8 per group. BV/TV: bone volume per unit of total volume; Tb.Th: trabecular thickness; Tb.N: trabecular number; Tb.Sp: trabecular separation; Tb.Pf: trabecular bone pattern factor; SMI: structure model index.

**Table 4:** Cortical bone microarchitecture in the tibial diaphysis.

Parameters (unit)	Control	GH	0.2% HT042	0.6% HT042
Tt.Ar (mm^2^)	3.853 ± 0.207	4.075 ± 0.204^*∗*^	3.888 ± 0.232	4.105 ± 0.326^*∗*^
Ct.Ar (mm^2^)	3.391 ± 0.197	3.604 ± 0.180^*∗*^	3.410 ± 0.222	3.627 ± 0.306^*∗*^
Ma.Ar (mm^2^)	0.463 ± 0.021	0.471 ± 0.032	0.478 ± 0.018	0.477 ± 0.028
Ct.Ar/Tt.Ar (%)	87.978 ± 0.564	88.440 ± 0.490	87.683 ± 0.621	88.340 ± 0.641
Ct.Th (mm)	0.412 ± 0.022	0.420 ± 0.017	0.393 ± 0.022	0.418 ± 0.024

Values are expressed as the mean ± SD. ^*∗*^*p* < 0.05  versus control group. *n* = 7-8 per group. Tt.Ar: total cross-sectional area; Ct.Ar: cortical bone area; Ma.Ar: medullary area; Ct.Ar/Tt.Ar: cortical area fraction; Ct.Th: cortical thickness.
